# The association between students’ confidence and ability to modulate spinal manipulation force–time characteristics of specific target forces: a cross-sectional study

**DOI:** 10.1186/s12998-024-00557-w

**Published:** 2024-11-11

**Authors:** Casper Nim, Nicole Smith, David Starmer, Simon Wang, Grand Choi, Akram Alayed, Jomana AlShareef, Angela Gnjatic, Keegan Sloan, Kitlyn Wong, Martha Funabashi

**Affiliations:** 1 Medical Research Unit, Spine Centre of Southern Denmark, University Hospital of Southern Denmark, Oestre Hougvej 55, 5500 Middelfart, Denmark; 2 Department of Regional Health Research, University of Southern Denmark, Odense, Denmark; 3 Department of Sport Science and Clinical Biomechanics, University of Southern Denmark, Odense, Denmark; 4 Canadian Memorial Chiropractic College, Toronto, ON Canada; 5 Department of Chiropractic, Université du Québec À Trois-Rivières, Trois-Rivières, Canada

**Keywords:** Spinal manipulation, Force-sensing table, Confidence, Teaching, Feedback

## Abstract

**Background:**

Spinal manipulative therapy (SMT) is a guideline-recommended care for musculoskeletal pain taught in various undergraduate programs. Visual feedback through force-sensing tables can improve modulation of SMT force–time characteristics and, potentially, students’ confidence, both factors important for clinical competence and patient outcomes. However, it is unclear if a link exists between students’ confidence and ability in SMT force–time modulation. We aim to investigate this relationship and whether it was moderated by experience.

**Methods:**

This cross-sectional study recruited first- to third-year Canadian Memorial Chiropractic College students. Participants provided information about their confidence in performing SMT using different impulse forces of 200N, 400N, and 800N with a pre-established pre-load and a time-to-peak force < 150ms. SMT impulse forces of 200N, 400N, and 800N were targeted on a Human Analogue Mannequin positioned prone on a force-sensing table. We described the confidence levels and SMT force–time characteristics and assessed their association using linear mixed models. We re-ran the models interacting with SMT experience. The order of the three SMT impulse forces was randomly performed. Participants and outcome assessors were blinded to force–time characteristics recordings.

**Results:**

One-hundred-and-forty-nine participants provided usable data. Participants were confident in delivering 200N and 400N impulse forces. However, confidence decreased for 800N forces. Accordingly, participants performed impulse forces close to the 200N and 400N but had difficulty accurately modulating to 800N forces. A positive association was found between confidence and the ability to modulate their force–time characteristics, especially keeping the same pre-load force, keeping the time to peak force < 150ms, and providing the 800N impulse force. This association was not moderated by experience.

**Conclusions:**

Students were more confident in their abilities to perform lower SMT forces but lacked confidence in their abilities to perform higher (800N) forces. This aligned with their skills, as many struggled to apply 800N force. However, students who had higher confidence levels generally performed better overall. There was substantial variability in SMT force–time characteristics, which may have implications for adverse events and patient satisfaction. Some of this variability could be attributed to students’ confidence. Thus, further investigations are necessary in undergraduate settings to implement and optimize these findings.

**Registration:**

https://osf.io/6f7d5

**Supplementary Information:**

The online version contains supplementary material available at 10.1186/s12998-024-00557-w.

## Background

Spinal manipulative therapy (SMT) is a hands-on intervention approach to treat spine-related pain. It is recommended by many clinical practice guidelines [1] and included in the new World Health Organization’s clinical guidelines for chronic low back pain [2]. Additionally, SMT applied to the thoracic spine appears to improve pain and disability in patients with thoracic spine pain and neck pain [3–7]. Previous studies emphasized the importance of applying specific force–time characteristics to improve SMT’s effectiveness and safety [8, 9]. Subsequently, several studies have focused on effective methods for teaching and learning SMT [10, 11].

Spinal manipulative therapy is a complex motor skill traditionally taught by having students replicate specific posture and body positioning and mimic specific body motions as demonstrated by experienced instructors while practicing SMT on each other [12]. However, the traditional approach appears to be associated with the risk of either new injuries or exaggeration of prior injuries in students [13, 14] and is arguably not ideal for students to fully comprehend SMT skills [12, 15]. Therefore, to theoretically facilitate the development of safer SMT skills, additional motor skill learning strategies, such as sensory and visual feedback, are being used [16, 17].

The importance and impact of feedback in refining students’ SMT motor skills have been emphasized by various studies. Specifically, a single feedback session can significantly improve the accuracy and consistency of achieving specific target peak forces [18]. Moreover, randomized controlled trials have shown that providing specific feedback on SMT force–time characteristics can significantly enhance both chiropractic and physiotherapy students’ ability to apply SMT with specific target force–time characteristics [21].

One way of providing students with visual feedback on their SMT force–time characteristics is by using force-sensing table technology (FSTT®, Canadian Memorial Chiropractic College, Toronto, Ontario, Canada) [22]. The FSTT® was developed as a training tool to quantify SMT forces and provide students with immediate feedback regarding their SMT force–time characteristics [22]. Twenty-one teaching institutions worldwide have integrated the FSTT® into their curriculum to facilitate learning and training to modulate SMT force–time characteristics [23].

Combining the use of FSTT® with motor skills teaching strategies, such as direct instruction from a tutor, can potentially improve students’ ability to modulate their SMT force–time characteristics and their confidence in performance [24, 25]. Moreover, the visual feedback provided by FSTT® can act as a positive reinforcement when students are able to perform the targeted SMT force–time characteristics, contributing to enhancing their confidence in their SMT skills [24]. Increased confidence is crucial for early-career clinicians, as it is linked to good clinical practice [26]. Importantly, increased confidence has been shown to enhance competency in other healthcare fields [27–29]. Therefore, enhanced confidence during professional training might contribute to higher competency not only as students but also as future healthcare professionals [26, 30, 31]. Although we are unsure if using the FSTT® directly affects students’ confidence levels and their ability to modulate their SMT force–time characteristics, we know that feedback, especially when combined with other motor skill teaching strategies, can increase confidence in learning general motor skills. Moreover, self-practice can further enhance this effect [17]. Therefore, visual feedback tools like the FSTT® has the potential of facilitating SMT training. However, we need more research to determine if the use of FSTT® can increase SMT skills and how it is associated with other factors, such as students’ confidence [11, 32].

### Objectives

Our primary objective was to investigate if there was an association between chiropractic students’ ability in modulating SMT force–time characteristics to specific targets and their confidence in performing it. Secondarily, we assessed if this association was modulated by experience with FSTT®.

We hypothesized that there would be a positive association between students’ confidence and their ability to modulate their SMT force–time characteristics to specific targets and this was moderated by FSTT® experience.

## Methods

### Design

This study was conducted using a cross-sectional design and followed a pre-determined protocol that was uploaded to Open Science Framework (https://osf.io/6f7d5). We adhered to the STrengthening the Reporting of OBservational studies in Epidemiology reporting guidelines [33]. This study was reviewed and approved by the CMCC Research Ethics Board (REB approval #2311B02). All participants reviewed and signed an electronic informed consent prior to participating in the study.

### Setting and study participants

Our study was conducted at the Canadian Memorial Chiropractic College’s FSTT® laboratory in Toronto, Canada. First to third-year, students were recruited using a convenience sampling approach. Students with musculoskeletal issues that would interfere with their ability to modulate their SMT force–time characteristics were excluded from the study.

Recruitment was done through study posters and a sign-up sheet placed at the FSTT® laboratory. During the test days, class announcements were made inviting students to visit the laboratory after completing their academic activities.

### Data collection

#### Study procedure

Students were introduced to the study by CN and given a study information letter (Additional file 1) before providing their informed consent to participate. Participants self-reported the variables of interest (see below) on an iPad (Apple, California, USA). All questionnaires were conducted via the SurveyMonkey electronic data capture platform (Momentive Inc., California, USA) with a blocked view of the study setup in the laboratory.

**Fig. 1:**
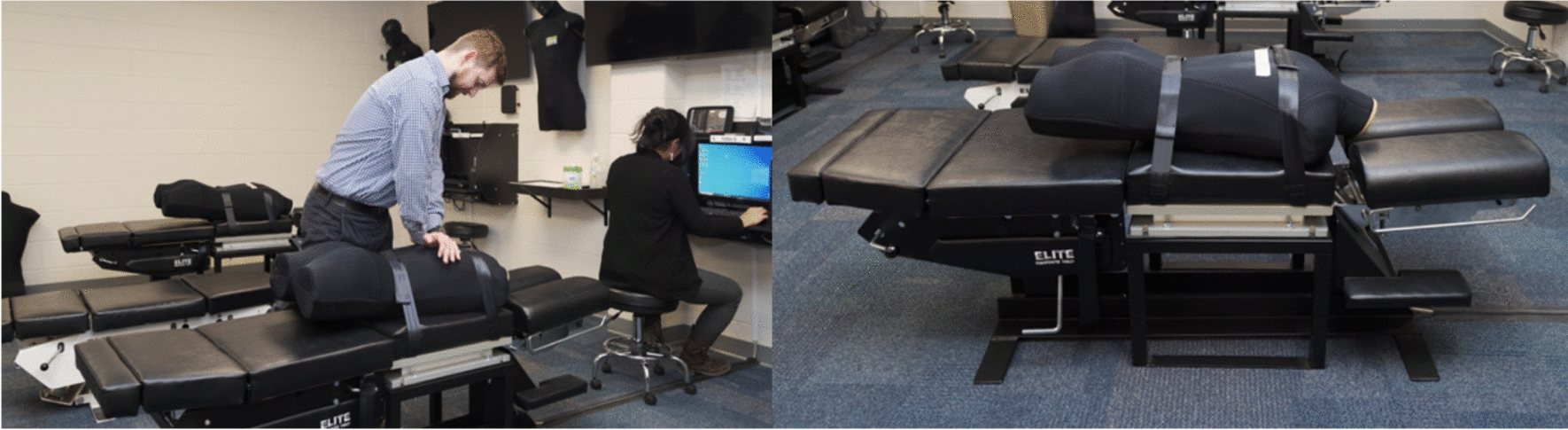
The study setup at the FSTT® laboratory

After completing the initial questionnaires, participants were directed to an FSTT® table by the first available investigator (NS, SW, AA, JA, AG, KS, KW, HA, MF). They were encouraged to choose a table that best suited their individual preference, such as height. A Human Analogue Manikin (HAM®, CMCC, Toronto, Ontario, Canada) was then placed in a prone position on the FSTT® and restrained by straps. The upper border of the shoulders was aligned with the edge of the table’s thoracic section, and a tape was placed at the mid-thoracic level (30cm from the upper border of the HAM®) to standardize the location of SMT application (Fig. 1).

Participants were first allowed to perform three practice thrusts using any SMT forces to get familiar with the study setup. Then, they were asked to define the preload force they judged appropriate for the HAM®. After that, the participants performed two posterior-to-anterior SMTs for each of the listed force–time characteristics.

- The pre-established pre-load (± 50N), followed by an impulse force of 200N (± 50N) with a time to peak force of < 150ms.
- The pre-established pre-load (± 50N), followed by an impulse force of 400N (± 50N) with a time to peak force of < 150ms.
- The pre-established pre-load (± 50N), followed by an impulse force of 800N (± 50N) with a time to peak force of < 150ms.

For each impulse force level, participants were allowed to choose their preferred hand contact (bilateral hypothenar, cross-bilateral hypothenar, or bilateral thenar) [33] but had to keep the same hand contact for both trials within each impulse force level. The order in which participants performed the impulse forces (200N, 400N, 800N) was randomized. This randomization was performed by MF using a random number generator in Microsoft Excel (Microsoft, Redmond, Washington, USA). The order was concealed using opaque envelopes packed by CN and MF, with each envelope containing a sequence of impulse forces (e.g., 200N, 800N, 400N) and given to the participant after they had consented to participate in the study. Participants only opened the envelope immediately before applying the first SMT. Investigators were kept unaware of the allocation sequence. In order to maintain blindness to the SMT force–time characteristics (graphics and values), the FSTT® software window was positioned on the screen so that only the record and save buttons were visible. A graph showing the thrust middle third unloading rate was visible to investigators. It was used to confirm that the FSTT® software identified a thrust; however, it did not inform the participant nor the investigator if target SMT force–time characteristics were performed. This blinding procedure was maintained from practice thrusts until the end of data collection, keeping the investigators and students blinded throughout the process.

##### Instrumentation

Three-dimensional SMT forces were measured and recorded using the FSTT® with a sampling rate of 2000 Hz. The FSTT® is composed of a modified Elite Stationary treatment table (Elite Chiropractic Tables, Jarvis, Ontario, Canada) with an embedded force plate (Advanced Mechanical Technology Inc., Watertown, Massachusetts, USA). The thoracic portion of the treatment table (with embedded force plate) was mechanically independent from the remainder of the table. The FSTT® readings were zeroed prior to each SMT application. Previous research has demonstrated excellent reliability of the FSTT® in measuring SMT force–time characteristics [22]. We used the FSTT® software with standardized algorithms to identify SMT force–time characteristics automatically.

#### Variables of interest

##### Demographic and anthropometric information

Participants provided self-reported information on the following characteristics:

- Sex [Male, Female]
- Gender [Man, Woman, Trans Man, Trans Woman, Gender fluid, Non-binary, Two Spirit, I do not identify with any, Other, Prefer not to answer]
- Age [Years]
- Height [Inches and reported in cm]
- Weight [Pounds and reported in kg]
- Hand preference [Right, Left]
- Year of study [1st, 2nd, 3rd]

##### Confidence of SMT force–time characteristics

Participants indicated their level of confidence in modulating SMT force–time characteristics to specific target forces using a 100mm electronic Visual Analogue Scale, where 0 corresponds to “not confident at all” and 100 corresponds to “completely confident” [24]. Participants provided confidence levels for:

- A pre-established preload (ability to perform the same preload [± 50N] for 200N, 400N, and 800N peak impulse forces)
- Peak impulse force (ability to perform 200N, 400N, and 800N [± 50N])
- Time to peak force (ability to perform 200N, 400N, and 800N impulse force in less than 150ms)

##### SMT application characteristics

We recorded the following parameters for each participant:

- Table chosen [1–7]
- Position to the treatment table [left, right]
- Hand contact [bilateral hypothenar, cross-bilateral hypothenar, bilateral thenar]

##### Time spent in the FSTT® laboratory

Data on participants’ experience was extracted from FSTT® total laboratory attendance records. One possibility was a tutor-led class, where students can attend and practice under faculty supervision [% of total laboratory attendance] (tutor-led lab). Additionally, students were able to sign in at the FSTT® laboratory during open practice hours and practice on their own. Each sign-in corresponds to one hour [hours] (open-practice lab). We extracted records corresponding to their attendance to the FSTT® laboratory in their current academic year (i.e., between June 2023 to January 2024).

#### SMT force–time outcomes

The ability to modulate SMT force–time characteristics to specific target forces was assessed through multiple outcomes.

- A pre-established preload force [N]. The force performed and held prior to the impulse force.
- Downward incisural point (DIP) [N]. The relinquishing of force application immediately prior to the impulse (i.e., the difference of force between the preload force and thrust onset force. [e.g., the participant applied a preload force of 200N but released the force and started the impulse at 150N, resulting in a difference of -50N]).
- Difference in peak impulse force from target impulse force [N] (e.g., the participant targeted 400N and performed an impulse force of 500N, resulting in a difference of + 100N).
- Time to peak impulse force [ms]. The time between the thrust onset force and peak impulse force.

Raw force plate data were automatically analyzed by the FSTT® software with standardized algorithms to identify SMT force–time characteristics of interest. Specific FSTT® data identified by the investigators were manually reviewed to ensure that the software correctly identified SMT force–time characteristics. When the automated analysis failed (e.g., failure to detect proper preload force and peak force points), relevant points of the force–time graph were manually identified by NS within the FSTT® software and re-calculated with consensus from NS, DS, GC, and MF.

### Statistical analyses

#### Sample size calculation

We did not have any pre-existing data to determine an exact sample size. However, we anticipated a moderate correlation for our primary objective with a high level of variability and approximately 5% of missing data due to erroneous measurements. Therefore, we aimed to include 120 students with an active effort to ensure balanced representation of participants across the three years of study.

#### Descriptive statistics

We tabulated demographic and anthropometric information using means and standard deviations or numbers and frequency as appropriate. We reported on SMT characteristics using numbers and frequency. All confidence and SMT force–time characteristics are presented using violin plots with embedded box plots to visually represent participants’ confidence levels stratified by each force–time target.

#### Associations between the ability to modulate SMT force–time characteristics and confidence

To illustrate the difference between the performed SMT force–time characteristic and its force–time targets, we also used violin plots with embedded box plots using the absolute differences between performance and target and incorporating the 50N accepted error rate. We conducted linear mixed regression models with the SMT force–time outcomes as the outcome variable and the confidence level interacting with the SMT force–time targets as fixed effects, with participant ID, table chosen, hand contact chosen, side of table chosen, and randomization sequence as random effects. We used the restricted maximum likelihood method to calculate model estimates. We used forest plots to visualize the results, which included mean slope estimates, 95% confidence intervals, and p-values calculated using a Wald t-distribution approximation. Instead of using the relative difference, we calculated the absolute difference between the performed and target preload and impulse forces. This is because the relative difference between the performed and target forces can be positive or negative depending on whether the performed force was lower or higher than the target force. Finally, each model was re-run, interacting with years of study and time spent in the FSTT® laboratory (tutor-led and open-practice lab analyzed separately). All models were checked for assumptions for normality of residuals by inspecting QQ-plots, linearity, and homogeneity of variance.

All data analyses were conducted using R version 4.3 and R-Studio version 2023.12 [34], utilizing the *Tidyverse* programming language [35]. Modeling was executed using the *lme4* [36] and *LMERtest* packages [37].

### Deviations from the protocol

We had to make minor amendments to the protocol following the study: (1) We were unable to include the 3rd-year students during the first period of data collection, which led us to increase our sample size from 120 to 150 participants to ensure a balanced distribution of first to third-year students. (2) We made some changes to our analytical approach. (3) We revised the classification of force modulation since allowing an error of ± 50N resulted in significant zero inflation, therefore, we used the raw values to describe our findings. (4) We assessed each target force independently and used the mean of the two SMT trials, rather than the second trial to account for potential measurement noise from the FSTT®.

## Results

### Descriptive statistics

We included 150 participants in January 2024. One participant had to be excluded as data indicated that the participant misunderstood the instructions and performed thrust at 200N, 400N, and 800N rather than following the randomization order (Additional file 2). As intended, there was a nearly equal distribution across the years of study. Participants were equally split between sexes, equally split between man and woman genders and two identified as other. The mean age of the participants was 24, with a limited age range (Table 1).

**Table 1:** Demographic and anthropometric information on 149 chiropractic students

Characteristic	N = 149
Year of study	
1st year	47 (32%)
2nd year	47 (32%)
3rd year	55 (37%)
Age	24 (3)
Sex	
Male	76 (51%)
Female	73 (49%)
Gender	
Man	75 (50%)
Woman	72 (48%)
Other/none	2 (1.3%)
Height [cm]	173 (15)
Weight [kg]	74 (20)
Hand dominance	
Right	135 (91%)
Left	14 (9.4%)

All seven testing stations were used at various times, ranging from 4 to 48 times. The majority of the participants (86%) preferred to apply the SMT on the left side of the table, and most participants used either a cross-bilateral hypothenar (49%) or bilateral hypothenar (38%) hand contact.

In total, there were 947 data trials of which 47 were recorded as erroneous trials (e.g., the FSTT® software did not identify a thrust) and the trial was repeated. Among the 900 valid data trials, automated analysis failed in 88 trials and relevant points of the SMT force–time graph were manually identified in all 88.

**Fig. 2:**
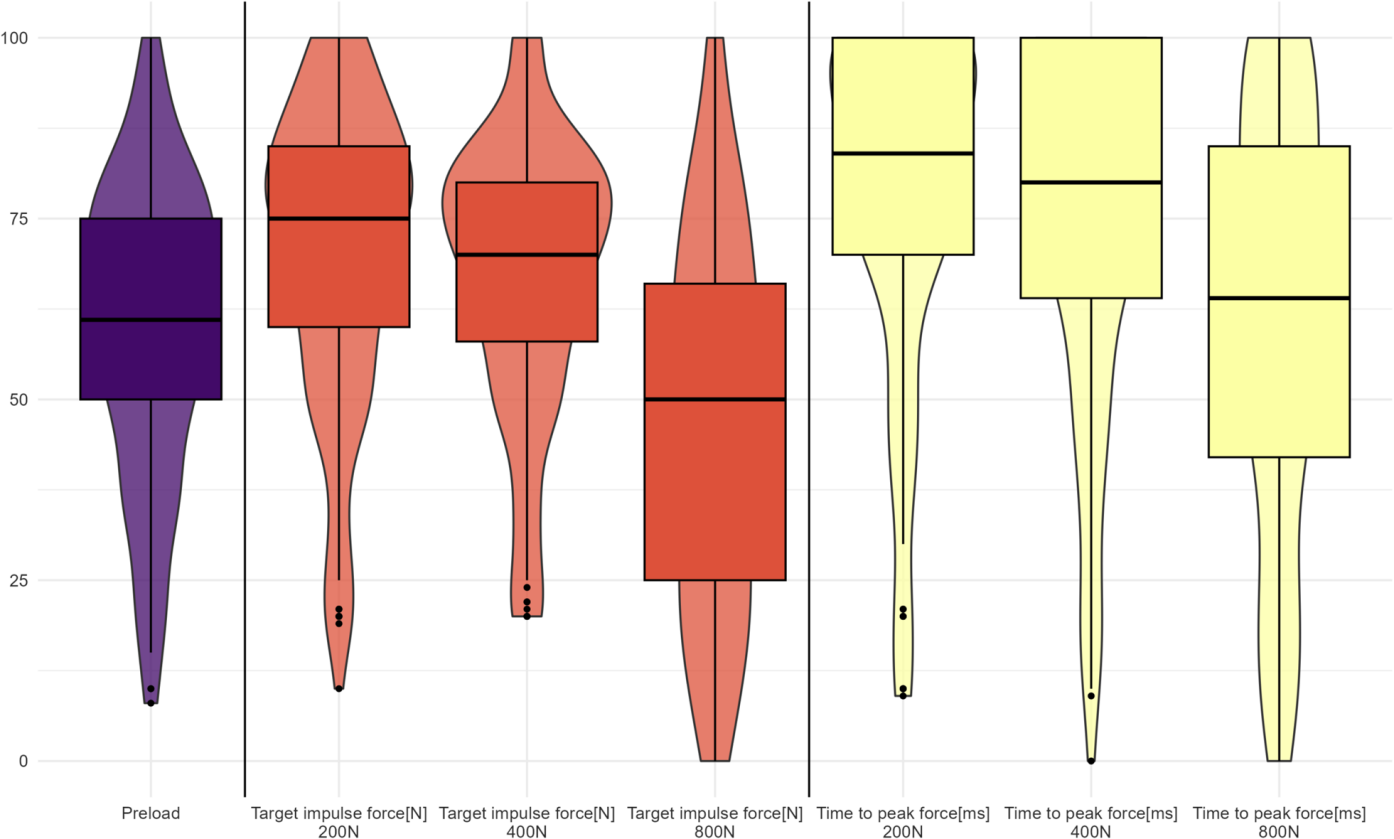
Students confidence levels of providing different SMT force–time characteristics across 200N, 400N, and 800N peak impulse forces

Participants generally felt confident in delivering impulse forces of 200N and 400N within a time to peak force of less than 150ms. However, when asked to deliver impulse forces of 800N, they felt less confident. They also felt less confident in maintaining a pre-established preload when applying all impulse forces. Nevertheless, more than half of all participants felt more than 50% confident in performing the specific SMT force–time characteristics (Fig. 2).

**Fig. 3:**
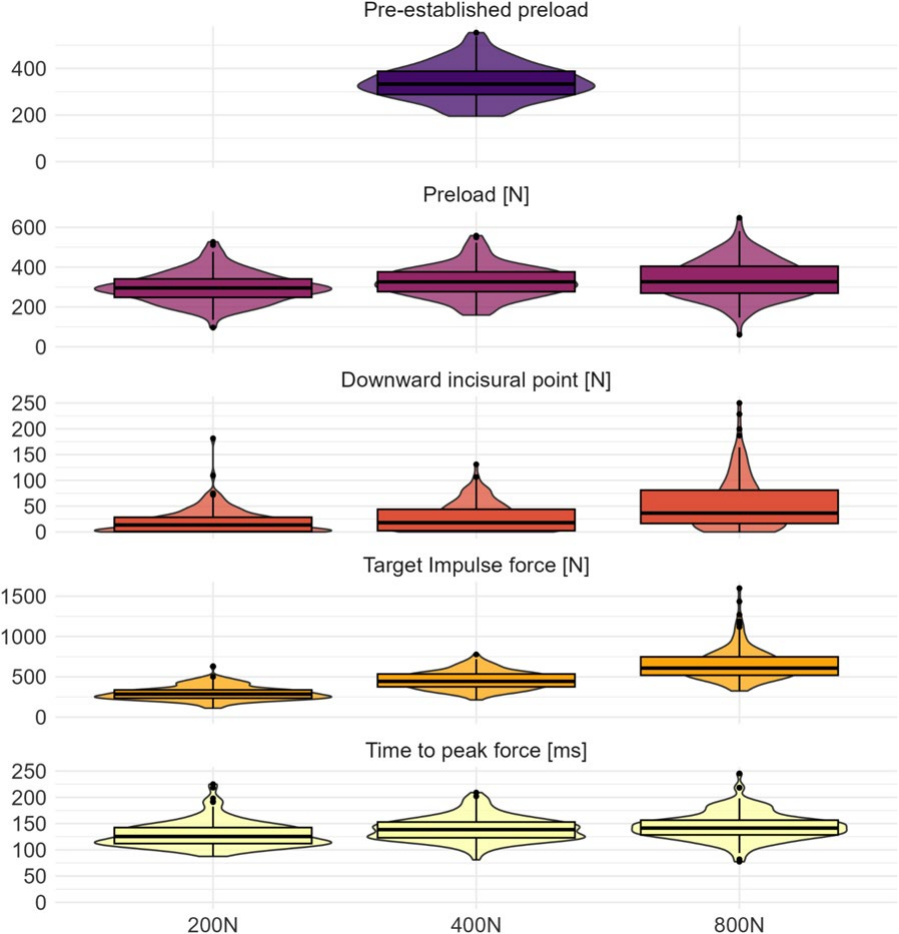
Students’ SMT force–time characteristics performed during their pre-established preload and across 200N, 400N, and 800N peak impulse forces. Y-axis values differ across plot facets

Participants performed wide ranges of forces for all SMT force–time targets. The pre-established preload primarily ranged between 300N and 400N, which was consistent with the preload provided across the performed impulse forces. We observed substantially more variability at the 800N impulse force target compared to 200N and 400N. However, most participants provided times to peak force of less than 150ms independent of the target force (Fig. 3).

### Associations between the ability to modulate SMT force–time characteristics and confidence

**Fig. 4:**
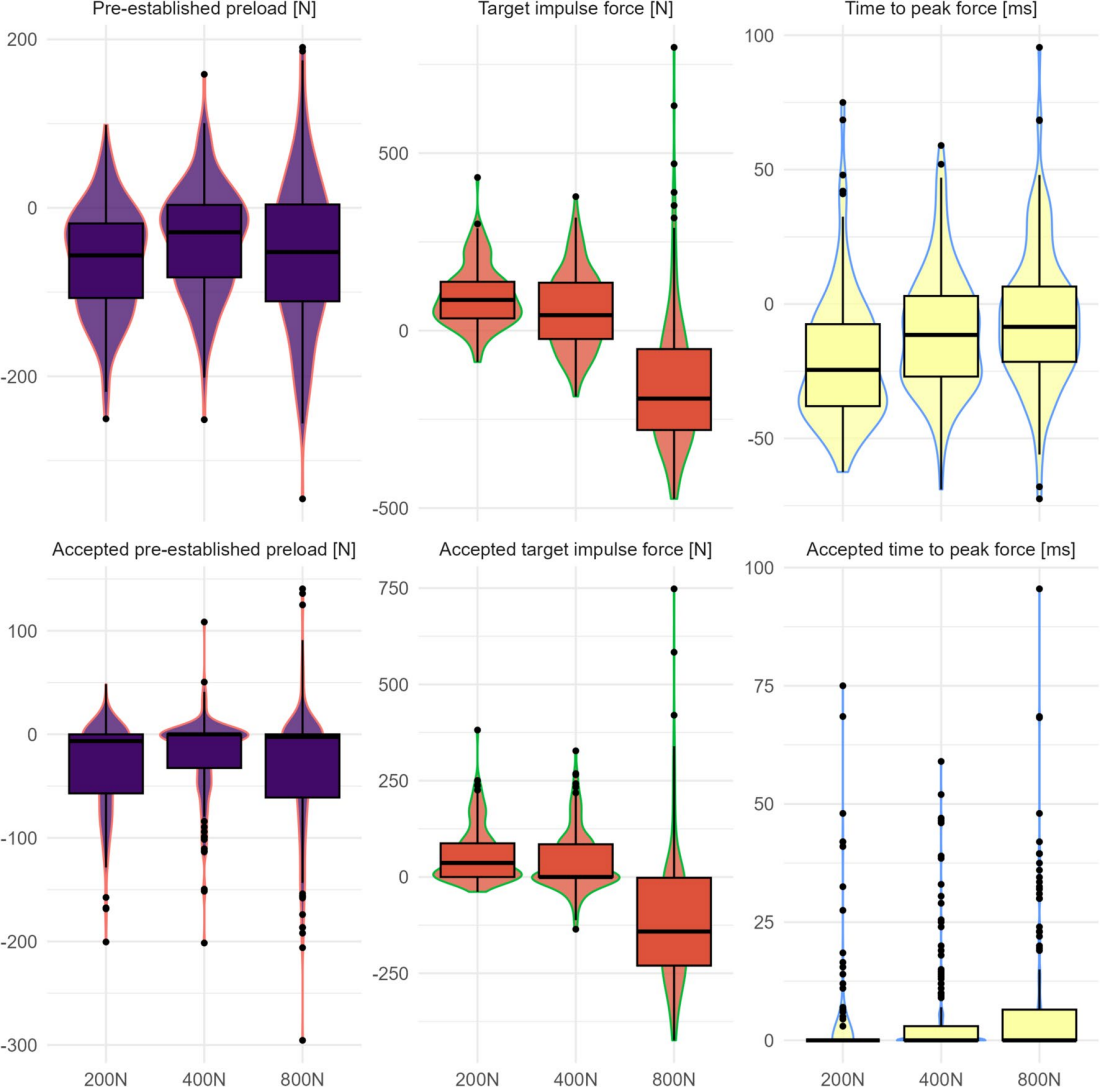
The difference between students performed SMT force–time characteristics and their target force–time characteristics. The top panel indicates raw differences, and the lower panel is standardized for the ± 50N error and < 150ms

The most substantial differences were observed at the 800N target impulse force, even after accounting for the ± 50N error. There were minimal differences in participants’ ability to perform the 200N and 400N target forces and, generally, performed impulse forces closer to the target forces. Moreover, most participants could apply the target impulse force in less than 150ms and consistently apply their pre-established preload. However, our results had considerable variability (Fig. 4).

**Fig. 5:**
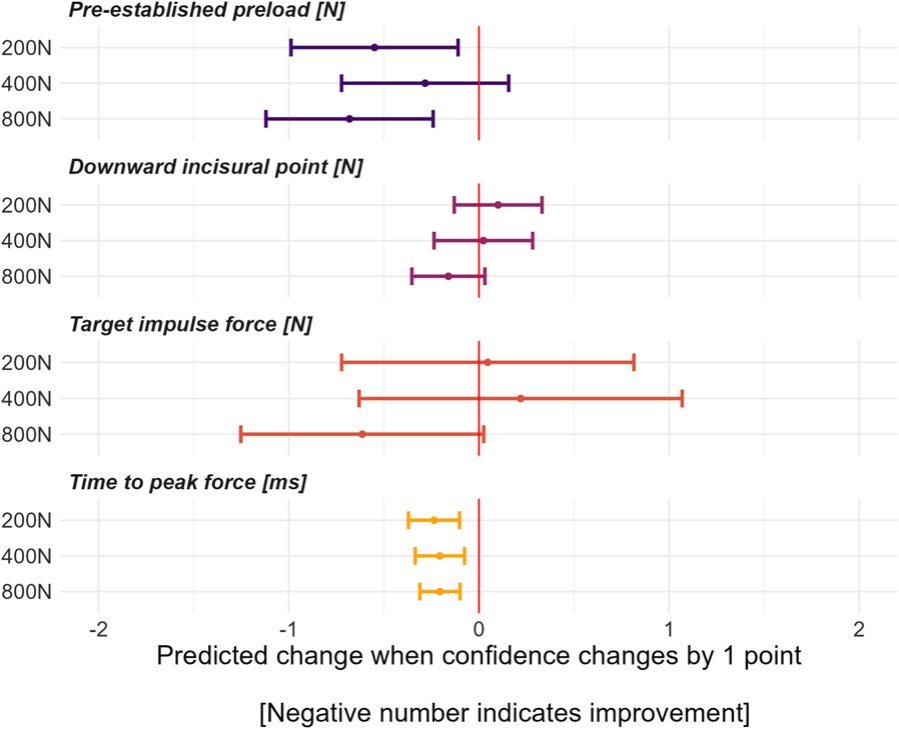
The association between students’ ability to modulate SMT force–time characteristics and their level of confidence in modulating SMT force–time characteristics. Positive slope estimates indicate that when student confidence increases, the differences between actual and target forces also increase. Negative slope estimates indicate that when student confidence increases, the differences between actual and target forces decrease (i.e., improve)

As the participants’ confidence increased, we observed an overall improvement in their ability to modulate their SMT force–time characteristics, especially the pre-established preload and time to peak forces. Although there was only a weak association between confidence and the DIP and target impulse forces, we noticed that when the target impulse force was 800N, students who performed forces that were closer to the target force were generally more confident (Fig. 5 and Additional file 3). The association between confidence and the ability to modulate SMT force–time characteristics was not substantially affected by either the year of study or the time spent in the FSTT® laboratory (either tutor or open lab), except for a minor difference among 3rd-year participants who had increased DIP forces when targeting 800N impulse force compared to 1st-year students and a minor interaction between confidence and time spent in tutor lab for the pre-established preload (Additional file 4). All models met the statistical assumptions.

## Discussion

### Summary of findings

Our study provides novel insights into the relationship between students’ confidence and specific SMT motor skills (i.e., ability to modulate SMT force–time characteristics). Students displayed higher confidence levels in their ability to perform SMT with impulse forces of 200N and 400N but lower confidence levels in performing impulse forces of 800N and maintaining a pre-established preload force across target impulse forces. Students’ confidence was aligned with their skills as many struggled to perform the higher target of 800N impulse force, even when accounting for an acceptable ± 50N error. We also observed a clear association between students’ confidence and SMT skills, particularly in preload force consistency, time to peak force, and delivering the most challenging impulse force—the 800N. Interestingly, this association was not affected by experience, as neither the years of studying nor time spent in the FSTT® laboratory moderated this association.

### Comparison with the literature

We observed significant variation in the SMT impulse forces performed across all target force–time characteristics. However, the lowest variability was seen when providing a time to peak force of less than 150ms. This outcome was expected and aligns with previous studies examining SMT force–time characteristics [38–40]. It is important to note that our results indicate that some of this variability in SMT force–time characteristics can be attributed to students’ confidence levels [41].

Neither the year of study nor time spent in the FSTT® laboratory moderated this association. Students in each year in the program have different exposures to the FSTT® laboratory, with 3rd-year students having more exposure and opportunities to practice and receive FSTT® visual feedback. Regardless of practice opportunities, however, participants in this study had limited experience with being assessed using such an objective tool. Although students are taught to deliver higher impulse forces (e.g., 800N), formal testing in the curriculum does not include forces of this magnitude. Therefore, the limited ability to perform the 800N impulse force observed could be related to limited practice, students not having developed the motor skills to perform such a target, and having less confidence. However, previous studies have measured applied forces exceeding 1,000N during thoracic spine SMT, indicating that higher forces are potentially used in practice [40]. This rationalizes why this was difficult but also why education on using high forces is indicated, as incorporating such targets may benefit some patients.

Students’ confidence and ability in modulating their SMT force–time characteristics may be influenced by other factors that are challenging to teach, assess, and evaluate. Some teaching strategies, like progressive laboratory feedback, have shown no improvement in their ability to perform specific SMT force–time targets [42]. Moreover, structured feedback like laboratory work may not necessarily lead to higher ability of modulating SMT force–time characteristics [41]. Self-controlled feedback may even be more efficacious than constant feedback [43]. This would be consistent with the lack of moderation found in time spent in the teaching laboratories.

It may not be surprising that the current year of study enrolled had limited impact in the relationship between students’ confidence and specific SMT skills. First, when teaching SMT force–time characteristics using visual feedback, improvement seems to happen early in their study, with only other biomechanical factors we did not measure developing over the years, such as body coordination [44]. Second, year of study and experience do not seem to influence the variability observed in SMT force–time characteristics [45, 46]. However, this might be different for different types of manual therapy techniques. Third, we are unsure how long the effects of teaching SMT force–time characteristics using visual feedback, such as the FSTT®, actually last [47], as this has not been researched sufficiently [16, 32]. However, preliminary work suggest retention of ability to modulate SMT force–time characteristics following six months of de-training [24].

### Teaching and clinical implications

There is wide variability of the force–time characteristics performed during SMT [40]. Arguably, individual patients may respond better to certain treatments and potential variations in those treatment applications. Subsequently, patient comfort has been reported to be an important factor of the patient experience [48, 49]. Additionally, altering SMT’s force–time characteristics has been observed to influence comfort and pain [50–52]. While ideal force–time characteristics that potentially influence clinical effects of SMT remain unknown, current educational approaches use visual feedback to train students to acquire the skills necessary to adapt their force–time characteristics to the individual needs of their patients, once in clinical practice.

Motor learning and retention of motor performance skills are complex and multifactorial [17]. Providing objective feedback on force modulation may facilitate and support students’ development of their motor strategies to master motor performance skills. Finding the balance between supporting their motor learning and making them overly reliant on feedback is challenging. Therefore, the high confidence and the limited ability to perform specific target impulse forces suggest a dependence of students on visual feedback to be able to modulate their forces. When deprived of it, their motor performance is affected.

Student confidence remains a relatively unexplored domain with unknown clinical impacts [26]. Even though our study focused on the SMT technique that students are most confident in, we did not find an association between confidence and all SMT skills. Therefore, confidence might be built based on factors other than the ability to modulate force–time characteristics, such as body anthropometrics, and should be explored further.

### Methodological considerations

We ensured the transparency and reproducibility of our research by designing the study and making the protocol, hypotheses, and analysis plan publicly available before enrolling participants. Although we had to make minor amendments to the protocol, we aimed to support the open research initiative. Moreover, we included a larger sample size than previous studies investigating FSTT®, which enabled us to draw more robust conclusions from our findings. However, we also conducted various testing of associations without controlling for multiple corrections. Therefore, we did not expressly rely on p-values but on confidence in estimate sizes [53].

The recruitment of participants primarily came from inviting students to participate during or following academic activities; this may result in selection bias as students with a higher interest in FSTT® and SMT, in general, might have been more inclined to participate. They will likely show more confidence and a better ability to modulate their SMT force–time characteristics than less interested students. The large sample size also came with the cost of having many testers and different test stations. To maintain consistency, we used standardized procedures, including a specific script for testers to follow, and provided standardized information material to the participants. Additionally, we used identical mannikins across all testing stations, as recommended for testing SMT force–time characteristics [54]. Still, with many students being tested simultaneously, not all students may have had the most optimal choice in selecting a specific table that fitted their preference and had to select whatever was available. This is important as it might increase the difficulty of completing the assigned task, as increasing task difficulty is associated with more SMT force–time characteristic variability [55], potentially resulting in ecological bias and skewing our results towards a null finding. Thus, we included the table chosen as a random factor within our modeling to adjust for any such potential. Another potential source of ecological bias was that the students had to perform the force without visual feedback from the FSTT®. The students are used to having visual feedback readily available when practicing their skills, and visual feedback has been shown to increase accuracy in performing specific force targets [56]. We considered this potential bias a necessary risk to ensure clinical translatability.

The SMT force–time characteristics at the table interface measured and recorded by the FSTT® have been found to be different from the applied forces [57, 58]. So caution should be used as the SMT force–time characteristics reported in this study may not represent forces applied in clinical practice. Additionally, although the standardized algorithms to identify SMT force–time characteristics within the FSTT® software are generally accurate, the automated analysis can fail to detect proper preload and peak force points. While we carefully inspected and identified trials that needed manual identification of points, a few specific trials may have been missed, potentially adding to the variability in SMT force–time characteristics observed in this study.

## Conclusion

We found that students were more confident when targeting lower SMT impulse forces of 200N and 400N but lacked confidence with 800N forces. This aligned with their abilities, as many students struggled to perform an 800N force—however, students with higher confidence levels generally were able to perform forces closer to the target level. Like many prior studies using students, our study also revealed significant variability in modulation SMT force–time characteristics to specific targets, which could have implications for adverse events and patient satisfaction. Some of this variability can be attributed to the students’ confidence levels. Consequently, further investigations are necessary in undergraduate educational settings to implement and optimize these findings. For example, the ideal level of feedback to optimize motor learning, while ensuring appropriate levels of confidence when modulation SMT force–time characteristics.

## Supplementary Information

Additional file 1.

## Data Availability

Data is available upon reasonable request, when our pre-planned analyses have been completed, please contact data responsible at Canadian Memorial Chiropractic College Martha Funabashi (mfunabashi@cmcc.ca).

## Abbreviations

SMT: Spinal manipulative therapy
FSTT®: Force-sensing table technology
HAM®: Human analogue mannikin
DIP: Downward incisural point
