# Supplementary material for: The association between students’ confidence and ability to modulate spinal manipulation force–time characteristics of specific target forces: a cross-sectional study
Source: Chiropr Man Therap. 2024 Nov 11;32:34. doi: 10.1186/s12998-024-00557-w (PMC11552172; doi:10.1186/s12998-024-00557-w)
Supplement: Supplementary file 1 — Additional file 1. [file 12998_2024_557_MOESM1_ESM.docx]

The association between students’ confidence and ability to modulate spinal manipulation force-time characteristics of specific target forces: a cross-sectional study

Additional files

# Additional file 1

## Information Letter

### Study Title: Association between SMT force-time modulation of specific target forces and students’ confidence in delivering SMT

**Research Team:**

- Dr. Martha Funabashi
  Canadian Memorial Chiropractic College - mfunabashi@cmcc.ca
- Dr. Casper Nim
  University of Southern Denmark – casper.nim@rsyd.dk
- Dr. David Starmer
  Canadian Memorial Chiropractic College - dstarmer@cmcc.ca
- Dr. Simon Wang
  Canadian Memorial Chiropractic College - swang@cmcc.ca

### Purpose of the Study

To determine if there is an association between students’ ability to modulate their SMT force-time characteristics to specific target forces and their confidence in delivering SMT.

### Procedures Involved in this Study and Time Commitment

You will be asked to come to the FSTT® Laboratory at the Canadian Memorial Chiropractic College for data collection. You will be required for 1 unique session, which should take approximately 15 minutes in total.

This session will include:

#### Procedures

- Explanation of the study and the technique that you will be asked to perform on a manikin.
- Completing an intake form, providing your demographic and anthropometric information.
- Completing a questionnaire indicating your level of confidence in modulating SMT force-time characteristics to specific target forces.

If you agree to participate:

- You will be asked to perform posterior-to-anterior thoracic SMTs to a manikin on the FSTT® in the following steps:
  - Define the preload force magnitude you judge to be appropriate.
  - Apply 2 SMTs for each of the following force-time characteristics:
    - The pre-defined preload (±50N), 200N (±50N) impulse with a time to peak <150ms.
    - The pre-defined preload (±50N), 400N (±50N) impulse with a time to peak <150ms.
    - The pre-defined preload (±50N), 800N (±50N) impulse with a time to peak <150ms.
  - The order in which you will be asked to apply either the 200N, 400N, or 800N will be randomized.
  - We will then extract how long you have spent in the FSTT® Lab to see if this will influence the association between SMT force-time characteristics modulation to specific target forces and confidence in delivering SMT.

### Potential Risks and Associated Safeguards

- You may experience discomfort during the SMT application. You may discontinue at any time without penalty, especially if you are experiencing discomfort. If you feel uncomfortable at any point throughout the testing protocol, let the investigators know and they will discontinue the test.
- Although unlikely, muscle and ligament strain are potential risks with performing thoracic manipulation. Typically, this includes Grade 1 strains, which will likely resolve itself over the course of a few days with rest and minor care. While the investigators have taken reasonable precautions to minimize risk, in the unlikely event of an injury or emergency during testing, you will be immediately referred for evaluation to the chiropractic clinic located on campus, and standard emergency procedures will be followed should they be required.

### Changing Your Mind about Participation

You may withdraw from the study at any time without any penalty. To do so, indicate this to the investigators by saying, “I no longer wish to participate in this study”.

### Potential Benefits of Participation

Information obtained from this research may help inform the impact of using force feedback (such as the FSTT®) on students’ confidence in delivering SMT.

### Confidentiality

Each participant will be assigned a unique identification code consisting of three numbers. Only the principal investigator of this study will have access to this code and the data related to this study. Any identifiable information will be destroyed once data collection is complete. All unidentifiable data will be stored indefinitely on computer hard drives (password protected). Only the study investigators involved in the data analysis (MF and CN) will have access to the data. Data will remain anonymous for long term storage and may be used in future research (secondary analysis). All data will be analyzed in a combined manner and no individual data will be analyzed or presented at any time. Unidentifiable data may be used for secondary analysis.

### Concerns about Participation

We would like to assure you that this study has been reviewed by, and received ethics clearance through, CMCC’s Research Ethics Board (REB). However, the final decision about participation is yours. In the event you have any comments or concerns resulting from your participation in this study, please contact Mr. Mark Fillery at XXX.

### Questions about the Study

If you have any further questions or want any other information about this study, please feel free to ask the investigators. If you have additional questions at a later date, please contact Dr. Martha Funabashi at XXX or by e-mail at mfunabashi@cmcc.ca.

**Sincerely Yours,**

Dr. Martha Funabashi

## The script that outcome assessors used to instruct participants and to collect data

Participant arrives, is greeted by [investigator], and gets handed: - A sealed envelop with their study ID number on it and concealed randomization order inside - A tablet with the survey opened [investigator to input their Study ID in the first page of the survey]

Once survey is completed, participants can choose a FSTT they would like to collect data on and use that FSTT. [research assistant to accompany the participant to the chosen table]

**Data collection operator script**

“Ok, now we will do SMT collection. Just a reminder, we will first get you to define the preload you judge appropriate. Then, you will perform 2 thrusts with each force level: 200N impulse, 400N impulse and 800N impulse. For each thrust, go as fast as you can, with a target of a time to peak of < 150ms. You can use the hand contact you prefer, on the side you prefer. Are you familiar with the meaning of the impulse force, and time to peak?

You will do one thrust at a time and in a random order that is in the envelope you have. Please don’t show or tell me what order and what force you will be applying. You can warm up by performing up to 3 thrusts at any force you want to familiarize yourself with the mannequin on the table. You will not be allowed to see your force-time data. When you are ready, we will record 2 thrusts for each target force and I will ask you to move to the foot-end of the table in between recording trials.

Do you have any questions?”

**Data collection steps:**

**Initial Steps** 1. Ensure you are not able to see the force-time graph on the FSTT software. 2. Participant to choose what side of the table they prefer.

**Warm up** 3. Warm up (up to 3 thrusts of their choice – no feedback provided)

**Preload** 4. Establish preload: - Participant to find their preload - Initiate recording, wait for ding - Apply preload, wait for 1-2 seconds, release - What hand contact did you use? - Save trial in the “Confidence Study” folder as: Study ID_Table#_TableSide_HandContact_Preload Example: 079_4_R_Cross_Preload

**Trials** 5. Participant to walk to the foot-end of the table, open the envelope. 6. Walk to the preferred side of the table and get ready to apply the first thrust, trial 1 - Are you ready? - Initiate recording, wait for ding - Apply SMT - What hand contact did you use? - Save trial in the “Confidence Study” folder as: Study ID_Table#_TableSide_HandContact_ThrustTrial# Example: 079_4_R_Carv_A1 7. Participant to walk back to the foot-end of the table 8. Walk back to the preferred side and get ready to apply the first thrust, trial 2 - Are you ready? [Ensure they are using the same hand contact as trial 1] - Initiate recording, wait for ding - Apply SMT - What hand contact did you use? - Save trial in the “Confidence Study” folder

Repeat until all 3 levels of force were recorded (2 trials each)

Confirm the number of files – each participant (same Study ID) should have 7 files.

| What is being recorded | File name |
| --- | --- |
| Preload | Study ID_Table#_TableSide_HandContact_Preload |
| 1st thrust | Study ID_Table#_TableSide_HandContact_A1 |
|  | Study ID_Table#_TableSide_HandContact_A2 |
| 2nd thrust | Study ID_Table#_TableSide_HandContact_B1 |
|  | Study ID_Table#_TableSide_HandContact_B2 |
| 3rd thrust | Study ID_Table#_TableSide_HandContact_C1 |
|  | Study ID_Table#_TableSide_HandContact_C2 |

“Ok, that is it! Please drop the envelope with the thrust order in that box [point to box] and we are done!

Thank you very much for participating in our study!”

**Hand Contacts:** - Carver = Carv - Cross Bilateral Hypothenar = Cross - Bilateral Thenar = Bither

# Additional file 2

The student whose data we did not include our analyses, as we determined in consensus that the student provided forces in the following order: 200N, 400N, 800N rather than the randomized order: 800N, 400N, 200N.


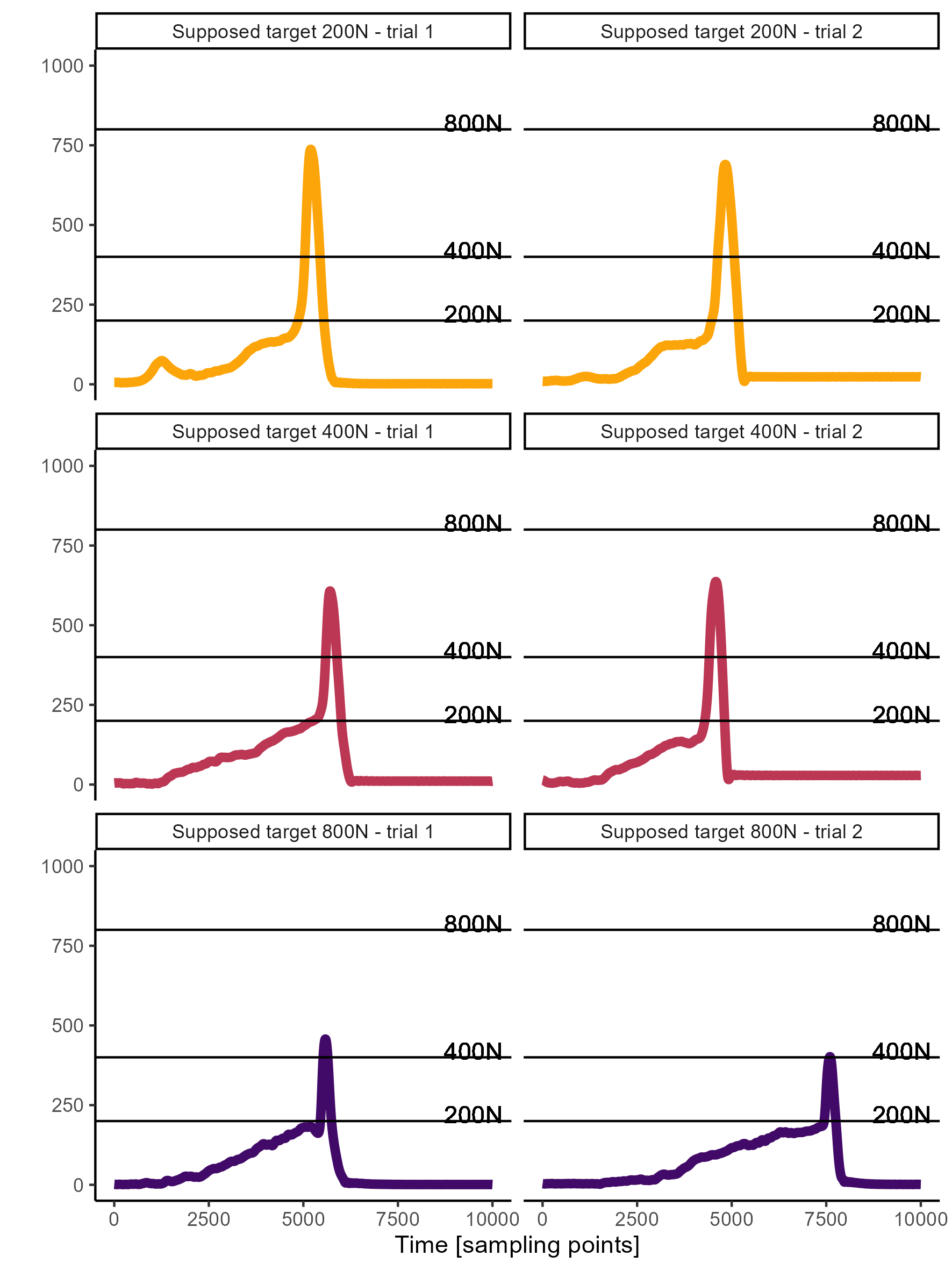


# Additional file 3

## The estimates from the model used for our primary analyses

Unadjusted models

|  | ***Preload*** | | | ***Downward insicural point*** | | | ***Peak impulse*** | | | ***Time to peak impulse*** | | |
| --- | --- | --- | --- | --- | --- | --- | --- | --- | --- | --- | --- | --- |
| **Characteristic** | **Beta** | **95% CI** | **p-value** | **Beta** | **95% CI** | **p-value** | **Beta** | **95% CI** | **p-value** | **Beta** | **95% CI** | **p-value** |
| **Confidence** | -0.55 | -0.98, -0.12 | **0.013** | 0.10 | -0.13, 0.33 | 0.39 | 0.05 | -0.71, 0.80 | 0.90 | -0.24 | -0.37, -0.10 | **<0.001** |
| **Target impulse** |  |  |  |  |  |  |  |  |  |  |  |  |
| *200N* | — | — |  | — | — |  | — | — |  | — | — |  |
| *400N* | -22 | -49, 3.9 | 0.094 | 16 | -6.4, 38 | 0.16 | -15 | -95, 65 | 0.72 | 7.5 | -3.3, 18 | 0.17 |
| *800N* | 27 | 1.2, 54 | **0.040** | 52 | 33, 70 | **<0.001** | 141 | 76, 205 | **<0.001** | 9.4 | -0.73, 20 | 0.069 |
| **Confidence * Target impulse** |  |  |  |  |  |  |  |  |  |  |  |  |
| *Confidence * 400N* | 0.27 | -0.14, 0.67 | 0.20 | -0.08 | -0.39, 0.23 | 0.62 | 0.17 | -0.95, 1.3 | 0.76 | 0.03 | -0.10, 0.17 | 0.65 |
| *Confidence * 800N* | -0.13 | -0.54, 0.28 | 0.53 | -0.26 | -0.54, 0.02 | 0.068 | -0.66 | -1.6, 0.32 | 0.19 | 0.03 | -0.10, 0.16 | 0.64 |

# Additional file 4

## The estimates from the model adjusted for i) year of study and ii) use of FSTT® laboratory

Moderated by Year of study

|  | ***Preload*** | | | ***Downward insicural point*** | | | ***Peak impulse*** | | | ***Time to peak impulse*** | | |
| --- | --- | --- | --- | --- | --- | --- | --- | --- | --- | --- | --- | --- |
| **Characteristic** | **Beta** | **95% CI** | **p-value** | **Beta** | **95% CI** | **p-value** | **Beta** | **95% CI** | **p-value** | **Beta** | **95% CI** | **p-value** |
| **Confidence** | 0.44 | -0.44, 1.3 | 0.33 | -0.09 | -0.43, 0.25 | 0.60 | 0.24 | -0.92, 1.4 | 0.69 | -0.32 | -0.46, -0.17 | **<0.001** |
| **Year of study** |  |  |  |  |  |  |  |  |  |  |  |  |
| *1st year* | — | — |  | — | — |  | — | — |  | — | — |  |
| *2nd year* | 21 | -63, 104 | 0.62 | -55 | -96, -15 | **0.008** | 9.5 | -127, 146 | 0.89 | -12 | -27, 4.0 | 0.15 |
| *3rd year* | -23 | -105, 59 | 0.58 | -27 | -68, 15 | 0.21 | 13 | -123, 149 | 0.85 | -7.6 | -25, 9.8 | 0.39 |
| **Confidence * Year of study** |  |  |  |  |  |  |  |  |  |  |  |  |
| *Confidence * 2nd year* | -0.08 | -1.4, 1.3 | 0.91 | 0.58 | 0.03, 1.1 | **0.037** | -0.21 | -2.1, 1.6 | 0.82 | 0.06 | -0.14, 0.26 | 0.53 |
| *Confidence * 3rd year* | 0.23 | -1.0, 1.5 | 0.72 | 0.31 | -0.26, 0.87 | 0.29 | -0.38 | -2.3, 1.5 | 0.70 | -0.02 | -0.24, 0.21 | 0.88 |
| **Target impulse** |  |  |  |  |  |  |  |  |  |  |  |  |
| *200N* |  |  |  | — | — |  | — | — |  |  |  |  |
| *400N* |  |  |  | 0.74 | -31, 32 | 0.96 | -65 | -183, 53 | 0.28 |  |  |  |
| *800N* |  |  |  | 26 | 0.01, 52 | **0.050** | 174 | 79, 270 | **<0.001** |  |  |  |
| **Confidence * Target impulse** |  |  |  |  |  |  |  |  |  |  |  |  |
| *Confidence * 400N* |  |  |  | -0.03 | -0.51, 0.45 | 0.91 | 0.67 | -1.1, 2.5 | 0.46 |  |  |  |
| *Confidence * 800N* |  |  |  | -0.07 | -0.50, 0.35 | 0.73 | -1.6 | -3.1, -0.06 | **0.042** |  |  |  |
| **Target impulse * Year of study** |  |  |  |  |  |  |  |  |  |  |  |  |
| *400N * 2nd year* |  |  |  | 41 | -11, 93 | 0.12 | 149 | -49, 346 | 0.14 |  |  |  |
| *800N * 2nd year* |  |  |  | 44 | 0.03, 89 | **0.050** | -29 | -188, 129 | 0.72 |  |  |  |
| *400N * 3rd year* |  |  |  | 29 | -23, 80 | 0.28 | 47 | -150, 244 | 0.64 |  |  |  |
| *800N * 3rd year* |  |  |  | 66 | 22, 109 | **0.003** | -88 | -247, 72 | 0.28 |  |  |  |
| **Confidence * Target impulse * Year of study** |  |  |  |  |  |  |  |  |  |  |  |  |
| *Confidence * 400N * 2nd year* |  |  |  | -0.35 | -1.1, 0.38 | 0.35 | -1.8 | -4.5, 0.99 | 0.21 |  |  |  |
| *Confidence * 800N * 2nd year* |  |  |  | -0.37 | -1.1, 0.31 | 0.29 | 0.81 | -1.6, 3.2 | 0.51 |  |  |  |
| *Confidence * 400N * 3rd year* |  |  |  | -0.16 | -0.90, 0.59 | 0.68 | -0.30 | -3.1, 2.5 | 0.83 |  |  |  |
| *Confidence * 800N * 3rd year* |  |  |  | -0.67 | -1.3, -0.01 | **0.046** | 2.1 | -0.36, 4.5 | 0.094 |  |  |  |

Moderated by Tutor lab (%)

|  | ***Preload*** | | | ***Downward insicural point*** | | | ***Peak impulse*** | | | ***Time to peak impulse*** | | |
| --- | --- | --- | --- | --- | --- | --- | --- | --- | --- | --- | --- | --- |
| **Characteristic** | **Beta** | **95% CI** | **p-value** | **Beta** | **95% CI** | **p-value** | **Beta** | **95% CI** | **p-value** | **Beta** | **95% CI** | **p-value** |
| **Confidence** | 3.9 | -0.03, 7.9 | 0.052 | 0.26 | -2.2, 2.7 | 0.83 | 3.0 | -4.8, 11 | 0.45 | -0.08 | -0.95, 0.78 | 0.85 |
| **Tutor lab** | 3.0 | 0.32, 5.8 | **0.029** | 0.64 | -1.2, 2.5 | 0.50 | 2.5 | -3.4, 8.4 | 0.41 | 0.48 | -0.28, 1.2 | 0.22 |
| **Confidence * Tutor lab** | -0.05 | -0.09, -0.01 | **0.029** | 0.00 | -0.03, 0.03 | 0.91 | -0.03 | -0.12, 0.05 | 0.45 | 0.00 | -0.01, 0.01 | 0.56 |
| **Target impulse** |  |  |  |  |  |  |  |  |  |  |  |  |
| *200N* |  |  |  | — | — |  | — | — |  |  |  |  |
| *400N* |  |  |  | 21 | -186, 228 | 0.84 | 603 | -167, 1,372 | 0.12 |  |  |  |
| *800N* |  |  |  | 93 | -86, 272 | 0.31 | 492 | -144, 1,128 | 0.13 |  |  |  |
| **Confidence * Target impulse** |  |  |  |  |  |  |  |  |  |  |  |  |
| *Confidence * 400N* |  |  |  | 0.71 | -2.3, 3.8 | 0.65 | -6.5 | -18, 4.8 | 0.26 |  |  |  |
| *Confidence * 800N* |  |  |  | -0.53 | -3.3, 2.3 | 0.71 | -5.4 | -15, 4.5 | 0.28 |  |  |  |
| **Target impulse * Tutor lab** |  |  |  |  |  |  |  |  |  |  |  |  |
| *400N * Tutor lab* |  |  |  | -0.05 | -2.3, 2.2 | 0.97 | -6.9 | -15, 1.6 | 0.11 |  |  |  |
| *800N * Tutor lab* |  |  |  | -0.47 | -2.4, 1.5 | 0.64 | -3.9 | -11, 3.1 | 0.28 |  |  |  |
| **Confidence * Target impulse * Tutor lab** |  |  |  |  |  |  |  |  |  |  |  |  |
| *Confidence * 400N * Tutor lab* |  |  |  | -0.01 | -0.04, 0.02 | 0.59 | 0.07 | -0.05, 0.20 | 0.25 |  |  |  |
| *Confidence * 800N * Tutor lab* |  |  |  | 0.00 | -0.03, 0.03 | 0.85 | 0.05 | -0.06, 0.16 | 0.35 |  |  |  |

Moderated by Open lab (hours)

|  | ***Preload*** | | | ***Downward insicural point*** | | | ***Peak impulse*** | | | ***Time to peak impulse*** | | |
| --- | --- | --- | --- | --- | --- | --- | --- | --- | --- | --- | --- | --- |
| **Characteristic** | **Beta** | **95% CI** | **p-value** | **Beta** | **95% CI** | **p-value** | **Beta** | **95% CI** | **p-value** | **Beta** | **95% CI** | **p-value** |
| **Confidence** | -0.38 | -0.81, 0.06 | 0.087 | 0.03 | -0.26, 0.32 | 0.84 | 0.23 | -0.73, 1.2 | 0.63 | -0.34 | -0.45, -0.23 | **<0.001** |
| **Open lab** | 0.43 | -0.76, 1.6 | 0.47 | -0.54 | -2.4, 1.3 | 0.56 | 1.1 | -4.8, 7.0 | 0.71 | -0.17 | -0.82, 0.48 | 0.61 |
| **Confidence * Open lab** | -0.01 | -0.03, 0.01 | 0.35 | 0.00 | -0.02, 0.03 | 0.67 | -0.02 | -0.09, 0.05 | 0.57 | 0.00 | -0.01, 0.01 | 0.86 |
| **Target impulse** |  |  |  |  |  |  |  |  |  |  |  |  |
| *200N* |  |  |  | — | — |  | — | — |  |  |  |  |
| *400N* |  |  |  | 16 | -12, 44 | 0.27 | -23 | -125, 80 | 0.66 |  |  |  |
| *800N* |  |  |  | 50 | 26, 73 | **<0.001** | 155 | 74, 236 | **<0.001** |  |  |  |
| **Confidence * Target impulse** |  |  |  |  |  |  |  |  |  |  |  |  |
| *Confidence * 400N* |  |  |  | -0.06 | -0.45, 0.33 | 0.77 | 0.27 | -1.1, 1.7 | 0.70 |  |  |  |
| *Confidence * 800N* |  |  |  | -0.17 | -0.51, 0.17 | 0.32 | -0.81 | -2.0, 0.39 | 0.18 |  |  |  |
| **Target impulse * Open lab** |  |  |  |  |  |  |  |  |  |  |  |  |
| *400N * Open lab* |  |  |  | -0.23 | -2.5, 2.1 | 0.84 | 0.98 | -7.7, 9.6 | 0.82 |  |  |  |
| *800N * Open lab* |  |  |  | -0.08 | -1.9, 1.7 | 0.93 | -1.3 | -7.5, 4.9 | 0.69 |  |  |  |
| **Confidence * Target impulse * Open lab** |  |  |  |  |  |  |  |  |  |  |  |  |
| *Confidence * 400N * Open lab* |  |  |  | 0.00 | -0.03, 0.03 | 0.98 | -0.01 | -0.12, 0.09 | 0.81 |  |  |  |
| *Confidence * 800N * Open lab* |  |  |  | -0.01 | -0.03, 0.02 | 0.54 | 0.01 | -0.07, 0.09 | 0.88 |  |  |  |
